# Can complement fix placental malaria?

**DOI:** 10.1186/s12916-021-02083-5

**Published:** 2021-09-10

**Authors:** Justin Y. A. Doritchamou, Patrick E. Duffy

**Affiliations:** Laboratory of Malaria Immunology and Vaccinology, National Institute of Allergy and Infectious Diseases, National Institutes of Health, Bethesda, USA

**Keywords:** Placental malaria, *Plasmodium falciparum*, Antibody, Complement, C1q, VAR2CSA, Vaccines

## The problem of placental malaria

Placental malaria (PM) is a deadly public health problem caused by the human parasite *Plasmodium falciparum*, and this scourge will get worse as existing control measures lose potency. Our understanding of PM pathogenesis suggests a vaccine is feasible, but first-generation candidates yielded only modest variant-specific activity in early trials. In this issue of *BMC Medicine*, Opi and colleagues provide evidence for a heretofore unrecognized mechanism of protective immunity, whereby the neutralizing activity of antibody against PM parasites is enhanced by fixing the complement component C1q [1].

Recent estimates [2] hold that up to 50,000 maternal deaths and 200,000 stillbirths result from PM in Africa annually, with additional mortality during infancy related to preterm and low birthweight deliveries*.* Despite public health advances including chemoprevention using intermittent sulfadoxine-pyrimethamine (SP) treatments, as well as insecticide-treated bednets, the terrible toll on mothers and their offspring continues. Deaths will increase as parasite resistance to SP intensifies and spreads.

The underlying cause of PM is well-established: *P. falciparum*-infected erythrocytes (IEs) adhere to receptors, mainly chondroitin sulfate A (CSA), and sequester in intervillous spaces (maternal blood) of the placenta, eliciting an inflammatory response associated with poor maternal and fetal outcomes. The parasite ligand for CSA, VAR2CSA, is a member of the *P. falciparum* erythrocyte membrane protein 1 (PfEMP1) family of variant surface antigens. Its large size and extensive sequence variation make VAR2CSA a challenging target for vaccine developers, and insights into protective antibodies and epitopes are needed to focus immunogen design.

Nature suggests the solution to the PM problem. Over successive pregnancies, pregnant women in areas of endemicity develop resistance and simultaneously acquire antibodies against placental IEs, including antibody that blocks IE binding to CSA [3] or mediates IE opsonization/phagocytosis [4]. These functional antibodies are believed to target VAR2CSA, and antibodies to VAR2CSA increase over successive pregnancies, but the association of VAR2CSA antibodies to improved clinical outcomes has been inconsistent [5].

## A new role for complement in serum anti-adhesion activity

In their study, Opi and colleagues examine additional antibody mechanisms that might contribute to PM immunity. The immunoglobulins IgG1 and IgG3 dominate the naturally acquired response to placental IEs and to VAR2CSA, and both subclasses fix complement, which prompted the authors to examine antibody for complement-dependent functional activities [1]. Using samples from a longitudinal cohort of pregnant women in Papua New Guinea, the authors first demonstrated that serum antibodies fix complement on the IE surface of CSA-binding parasites. Levels of antibody-mediated complement fixation increased with gravidity and also predicted lower risk of PM at delivery among the subset of women who were infected at enrollment. This seroepidemiology suggests complement fixation might play a role in protective immunity.

The team examined possible mechanisms by which complement might neutralize parasites. They found that serum antibodies that bound recombinant VAR2CSA domains can fix the complement components C1q and C3. Using the CSA-binding laboratory isolate CS2 that maintains VAR2CSA on its IE surface, however, the group observed no evidence that complement fixation led to the formation of membrane attack complex or to inhibition of growth in vitro. These findings echo earlier studies with IEs from non-pregnant individuals, where the absence of complement activity was attributed to complement regulatory protein on the red cell surface [6, 7]. Instead, and surprisingly, fixation of C1q enhanced serum anti-adhesion activity against CSA-binding IEs.

Levels of complement on IE were reduced after CS2 was engineered to block PfEMP-1 surface expression, further implicating VAR2CSA as a target. Taken together, the data support the idea that VAR2CSA antibodies fix complement which enhances their anti-adhesion activity against CSA-binding IE and thereby might prevent placental sequestration. Of note, modest levels of complement were still detected on IEs without surface PfEMP1, and therefore, other surface antigens may also be targets of complement-fixing antibody.

## The contribution of complement to protective immunity

In contrast to these new findings, a recent study of Ghanaian gravidae [8] observed complement fixation by serum antibody on recombinant VAR2CSA but not on the IE surface of CSA-binding IT4/FCR3 parasites (that are isogenic to the CS2 parasite studied here). Opi and colleagues confirmed C3 fixation on the IE surface of an additional maternal isolate from Papua New Guinea and also showed that serum antibody bound to recombinant VAR2CSA fragments from different alleles fixed C1q or C3. Nevertheless, studies are warranted in different geographical regions using diverse parasites to understand how host or parasite heterogeneity impacts complement-dependent functional activity, and different alleles of full-length recombinant VAR2CSA ectodomain [9] should be assessed as reagents to measure complement-fixing antibody.

Future studies will also benefit by enrolling women earlier in gestation, as Opi et al. suggest, since C1q-binding serum antibody did not predict protection in women without infection at enrollment, a majority of the study population. Capturing the many parasitemia events that appear early in second trimester might strengthen the associations beyond those seen in this study. Finally, larger studies or meta-analyses will be needed to discriminate the independent contributions made by anti-adhesion antibodies, complement-dependent anti-adhesion activity, opsonizing antibodies, and other immune measures, to protection from PM.

## Conclusions

Opi and colleagues describe a role for complement to enhance protective anti-adhesion antibody activity during PM. Thus, VAR2CSA vaccine activity may similarly be enhanced by inducing antibodies that fix complement. The human response to the VAR2CSA vaccine PRIMVAC was dominated by IgG1 and IgG3, but nevertheless, serum anti-adhesion activity measured in vitro was modest against different parasite variants [10]. Given the findings of Opi et al., antisera from past and future trials should henceforth be examined with careful attention to complement in the assays. Vaccine design and formulation should also consider the potential contribution of complement-fixing antibodies to protective efficacy, alongside the contributions of opsonizing/phagocytosing and anti-adhesion antibodies. Ultimately, vaccine trials might provide the clearest evidence for the individual and combined benefits of these different immune mechanisms for preventing PM.

## Data Availability

Not applicable
